# Explosive or Continuous: Incoherent state determines the route to synchronization

**DOI:** 10.1038/srep12039

**Published:** 2015-07-10

**Authors:** Can Xu, Jian Gao, Yuting Sun, Xia Huang, Zhigang Zheng

**Affiliations:** 1 Department of Physics and the Beijing-Hong Kong-Singapore Joint Centre for Nonlinear and Complex Systems (Beijing), Beijing Normal University, Beijing 100875, China; 2 Department of Mathematics and Physics, North China Electric Power University, Beijing 102206, China

## Abstract

Abrupt and continuous spontaneous emergence of collective synchronization of coupled oscillators have attracted much attention. In this paper, we propose a dynamical ensemble order parameter equation that enables us to grasp the essential low-dimensional dynamical mechanism of synchronization in networks of coupled oscillators. Different solutions of the dynamical ensemble order parameter equation build correspondences with diverse collective states, and different bifurcations reveal various transitions among these collective states. The structural relationship between the incoherent state and the synchronous state leads to different routes of transitions to synchronization, either continuous or discontinuous. The explosive synchronization is determined by the bistable state where the measure of each state and the critical points are obtained analytically by using the dynamical ensemble order parameter equation. Our method and results hold for heterogeneous networks with star graph motifs such as scale-free networks, and hence, provide an effective approach in understanding the routes to synchronization in more general complex networks.

Understanding the intrinsic microscopic mechanism of collective behavior of populations of coupled units has become a focus in a variety of fields, such as biological neurons circadian rhythm, chemically reacting cells, and even society systems^1^,^2^,^3^,^4^,^5^,^6^,^7^. In particular, the abrupt transition to spontaneous collective synchronization in Kuramoto-like networked oscillators has attracted much attention in the last decade. For example, it was reported that a particular realization of a uniform natural frequency distribution of oscillators with an all-to-all network topology leads to a discontinuous first-order phase transition to synchronization^8^. Furthermore, when the frequencies of nodes are positively correlated to the node’s degrees, an abrupt transition from the incoherent state to the synchronization in heterogenous networks takes place^9^. Such a phenomenon of the first-order phase transition was termed as explosive synchronization in literature, and this explosive synchronization has been observed in frequency-weighted networks, and electronic circuits^10^,^11^. Numerous efforts have been made to understand the explosive synchronization from different viewpoints such as the topological structures of networks and coupling functions among nodes^11^,^12^,^13^,^14^,^15^,^16^. Some significant analytical works were reported to investigate the mechanism of the first-order phase transition to synchronization based on the mean-field theory^17^,^18^. The fact that the key point in understanding the discontinuous synchronization transition is the analysis of the multi-stability of miscellaneous synchronous attractors in phase space, for example the hysteretic behavior at the onset of synchronization^19^. However it is difficult to get an analytical insight in a high-dimensional phase space, and a convincing understanding is still lacking.

It is our motivation in this paper to reveal the mechanism of synchronization transition, especially the explosive synchronization in networks with a star motif by analyzing in a low-dimensional complex ensemble order parameter space in terms of the Ott-Antonsen method. Different solutions of the dynamical ensemble order parameter equation build correspondences with diverse collective states, and different bifurcations reveal various transitions among these collective states. The structural relationship between the incoherent state and the synchronous state leads to different routes of transitions to synchronization, either continuous or discontinuous, the explosive synchronization is a touchstone in testifying our approach. We reveal that the explosive synchronization is attributed to the coexistence of the incoherent state either stable or neutrally stable and the attracting synchronous state. The hysteresis is determined by the basin of attraction of bistable state where the measure of each state and the critical points are obtained analytically by using the dynamical ensemble order parameter equation. The scenario is further applied to discussions of the first-order phase transition in generic scale-free networks.

## Results

### Star network without phase shift

In a heterogeneous network, such as a scale-free network, hubs play a dominant role. Hence a star motif with a central hub is a typical topology in grasping the essential property of the heterogeneous networks. By keeping oscillators on *K* leaf nodes with the same frequency *ω* and the hub with *ω*_*h*_, the equations of motion can be written as


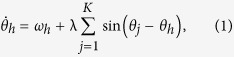



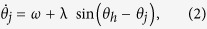


where 1 ≤ *j* ≤ *K*, *θ*_*h*_, *θ*_*j*_ are phases of the hub and leaf nodes, respectively, λ is the coupling strength. By introducing the phase difference *φ*_*j*_ = *θ*_*h*_ − *θ*_*j*_, Eqs (1) and (2) can be transformed to


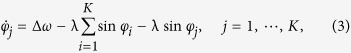


where Δ*ω* = *ω*_*h*_ − *ω* is the frequency difference between the hub and leaf nodes.

The synchronous state is defined as *φ*_*i*_(*t*) = *φ*_*j*_(*t*) ≡ *φ*(*t*) and 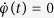, which can be solved from Eq. (3) as


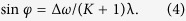


Since 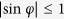, the synchronous state exists when 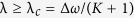. The synchronous state is found to be stable when λ ≥ λ_*c*_ by using linear-stability analysis. Further numerical computations reveal that the transition to the synchronous state is abrupt, and there is a hysteretic behavior at the onset of synchronization. 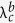 and 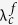 are the backward and forward critical coupling strengths respectively, where 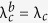 and 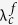 depends on initial states. The upper limit of 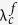 is denoted by 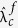. As 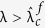, the synchronous state is globally attracting. The region between 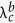 and 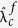 is the coexistence regime for the synchronous state and the incoherent state. The dynamic process of the synchronization transition depends crucially on the basin of attraction of each state^19^. However, it is hard to investigate the intermingling structure of these different attractors in the *K*-dimensional phase space {*φ*_*i*_, *i* = 1,2,…,*K*}, and till now only numerical works for not large *K* have been done. It is significant to find an analytical scheme to excavate the coexistence of synchronous and incoherent attractors and quantitatively reveal the mechanism of the discontinuous phase transition.

By introducing the order parameter


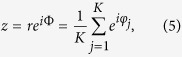


it is instructive to rewrite Eq. (3) as


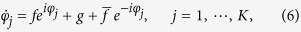


where *i* denotes the imaginary unit and 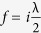, 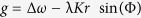. In terms of Watanabe-Strogatz’s approach, the phase dynamics of *K* nodes can be constructed from *K* constants {*ξ*_*j*_,1 ≤ *j* ≤ *K*} as


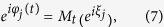


where


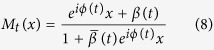


is a Möbius transformation^20^,^21^,^22^. By applying it to Eq. (6) one obtains


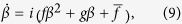



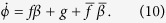


For the situation of thermodynamic limit *K* → ∞ and the uniform measure of phases, the evolution of *β*(*t*) and *ϕ*(*t*) in Eqs (9) and (10) can be separated. We thus get *β*(*t*) = *z*(*t*) and the equation of the order parameter as^23^


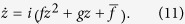


For a finite *K*, the fluctuation of the order parameter is of size 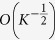. When 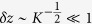, the order parameter *z* can be approximated by the one with infinite-*K* limit, i.e, 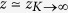. However, the typical size of the star network we are considering here is 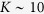. The fluctuation of this small system will be as large as 
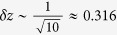
. It is not appropriate to approximate it with the one with infinite-*K* limit. Nevertheless, we can get the measure and the distribution of phases through an ensemble way. For an ensemble consisting of systems with same parameters and random initial conditions confined in an interval 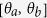, an ensemble order parameter is defined as


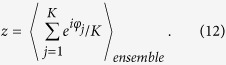


In the infinite limit of the number of systems in this ensemble, the Watanabe-Strogatz’s method is applicable. The dynamical equation of the ensemble order parameter of system is


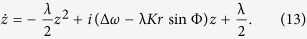


Eq. (13) describes the collective dynamics of Eqs (1) and (2) in terms of the ensemble order parameter. In the phase space of the ensemble order parameter, the synchronous state corresponds to a fixed point with *r* = 1 and a fixed phase Φ. All the other solutions of Eq. (13) represent various incoherent states. Some typical incoherent states include the splay state defined by *r* < 1 with a fixed phase Φ, the in-phase state defined by *r* = 1 with a periodic phase Φ(*t*) which means the phase of all the leaves are equal with a drifting hub and the neutral state defined by time-periodic *r*(*t*) and Φ(*t*). The transitions from these states to synchronization correspond to different scenarios of collective behaviors.

Eq. (13) can be rewritten in cartesian coordinates *z* = *x* + *iy* as


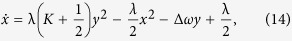



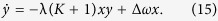


Eqs (14) and (15) are invariant under the time-reversal transformation (*t*,*x*) → (−*t*,−*x*). This implies the quasi-Hamiltonian property of Eqs (14) and (15)^24^, where the phase volume in the vicinity of any periodic orbits is conserved.

In the phase space of the ensemble order parameter, the natural boundary of the order parameter is *x*^2^ + *y*^2^ = 1. A fixed point is determined by the intersection of nullclines 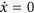 and 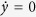 within the boundary. When the coupling λ is small enough, there is only one fixed point, which is neither an attractor nor a repellor for they should appear in pairs. Hence the incoherent state are neutrally stable periodic orbits around the fixed point. This can be verified by using the linear stability analysis.

**Figure 1:**
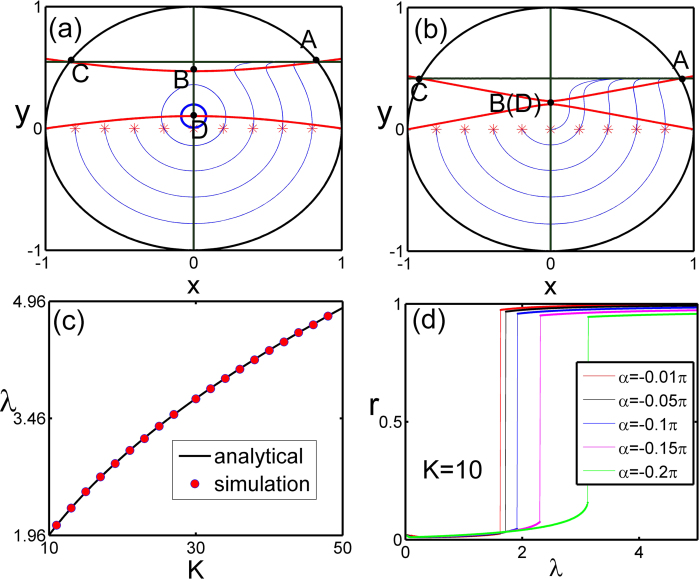
The ensemble order parameter phase plane of Eq. (5) with Δ*ω* = 9, *K* = 10 (**a**) λ = 1.5, (**b**) λ = 1.9. Red lines are 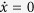, and black 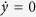. The intersections of them are fixed points A-D. Different initial values with trajectories are marked by ‘*’. (**c**)The upper limit of forward critical coupling strength in Eq. (16). (**d**) The forward continuation diagrams for star graphs of different *α*.

In the bistable regime, as shown in Fig. 1(a), the nullclines 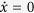 and 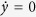 have four intersections labeled by **A**-**D** with **A** an attractor, **C** a repellor, **B** a saddle and **D** a neutrally stable point. Any orbit crossing the nullcline **A**-**B**-**C** will eventually fall to **A**, and others will hold the property as periodic orbits. It is clear that the stable fixed point **A** corresponds to the synchronous state.

As λ increases, points **D** and **B** close to each other and eventually collide at a critical coupling, as shown in Fig. 1(b), and the synchronous state becomes globally attractive. This critical coupling corresponds to the upper limit of 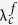, which can be determined as


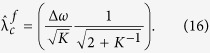


**Figure 2:**
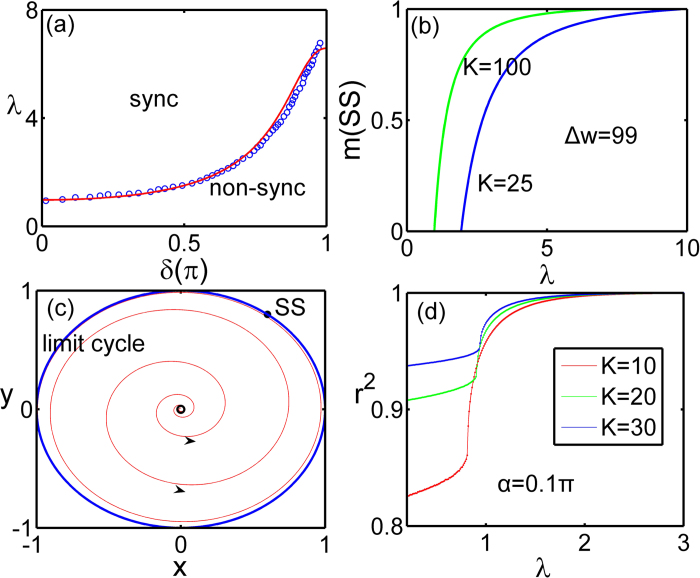
(**a**) Synchronization boundary line Eq. (18) with the initial distribution of phase are randomly drawn from interval [−*δ*,*δ*]. (**b**) The measure of the synchronous state against the coupling strength for different *K*. (**c**) The ensemble order parameter phase space for 0 < *α* ≤ 0.5*π*, the limit cycle corresponding to the splay state, and the fixed point corresponding to the synchronous state (SS). (**d**) The order parameter against the corresponding coupling strength with different sizes.

The analytical curve and numerical results are given in Fig. 1(c). An approximation of this result was previously estimated as 
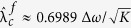
19, but this estimation is only the limiting case of Eq. (16) for large *K* where 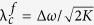. Moreover, the exact critical coupling strength which depends on different initial conditions^19^ could be analytically obtained. When the initial distribution of the phase difference between the leaves and the hub are randomly drawn from an interval 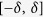, the initial order parameter is


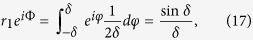


which means (*x*_0_ = sin *φ*/*δ*, *y*_0_ = 0). From the analysis above, the λ − *δ* relation is


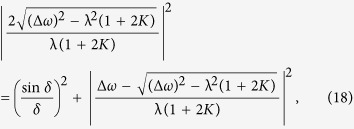


which is represented in Figs. 2(a).

It is important to compute the measure of the synchronous state in phase space when the synchronous state and the incoherent state coexist. The measure is defined as *m*(SS) = *S*_*syn*_/*S*_0_, where *S*_0_ and *S*_*syn*_ are the volume of the whole phase space and the volume of the basion of attraction of the synchronous state respectively. This can be analytically obtained in the ensemble order parameter space as *S*_*syn*_ ≈ *π*(1 − (*x*_B_ − *x*_D_)^2^) and *S*_0_ = *π*, where (*x*_B_,*y*_B_) and (*x*_D_,*y*_D_) are the coordinates for points **B** and **D** respectively. Therefore the measure of the synchronous state is


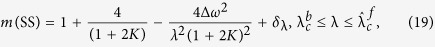


where *δ*_λ_ is the correction factor


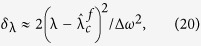


bounded by 1/*K*. When 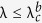, *m*(SS) and when 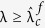, *m*(SS) = 1. In Fig. 2(b), *m*(SS) vs λ is shown. When *K*→∞, the measure can be approximated by *m*(SS) ≈ 1 − λ^−2^. Moreover the competition between the incoherent state and the synchronous state is represented in Fig. 2(b), with the increasing of coupling strength, the basin of attraction of the synchronous state is increasing while the incoherent state is decreasing. At the upper limit of the forward critical point, the synchronous state is globally attracting and the incoherent state will disappear. In addition, an alternative way for obtaining the critical coupling points is provided by the mean-field theory^18^ (we refer the interested reader to the Supplementary Information for the details of the derivations). We emphasize that, although the dynamical ensemble order parameter equation is successful in dealing with the identical leaves on star graph, it may fail for the random natural frequency distribution of the leaves. The latter case should refer to the mean-field method in^18^.

### Star network with a phase shift

The above results indicate that the ensemble order parameter approach can successfully describe the collective dynamics of coupled oscillators, and the dynamical ensemble order parameter equation provides an exact description in revealing the transitions, coexistence and competitions between incoherent and synchronous states. However, the quasi-Hamiltonian property of system Eqs (14) and (15) should be a specific case depending crucially on the coupling function. Therefore, it is significant to extend the dynamical ensemble order parameter approach to more general cases by adopting the Kuramoto model Eqs (1) and (2) with a phase shift^25^,^26^:


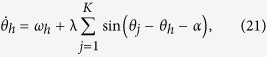



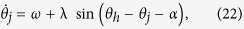


where −*π*/2 ≤ *α* ≤ *π*/2 is the phase shift, with *α* = 0 corresponding to the case of Eqs (1) and (2). When *α* = 0,±*π*/2 the equation is time reversible, and they divide the parameter space *α* into two dynamical regimes (−*π*/2,0) and (0,*π*/2).

By introducing *φ*_*j*_ = *θ*_*h*_ − *θ*_*j*_, Eqs (21) and (22) are transformed to


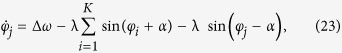


the phase locked state is a fixed point in the phase difference space, which reads
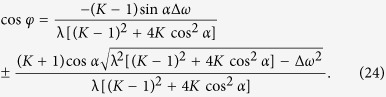
Since cos *φ* is real, the condition for existence of the synchronous state can be obtained similarly as


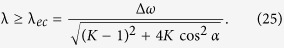


For local stability analysis of the phase locked state, the Jacobian matrix is calculated,


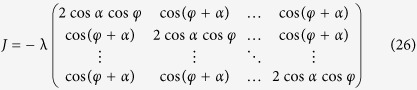


with *K* − 1 equal eigenvalues given by
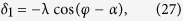
and one eigenvalue
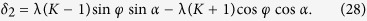
Through the analysis of the sign of eigenvalues *δ*_1_ and *δ*_2_, we get the stability conditions for the synchronous state, as


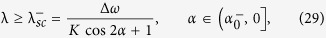



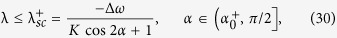


where 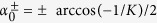. When 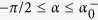 the synchronous state is always unstable, and when 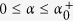 the synchronous state is always stable.

To get more information about the system, the dynamical ensemble order parameter equation for the system is constructed as,
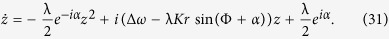
When λ is small enough, Eq. (31) always has one fixed point within the boundary in phase space (*x*,*y*). When *α* = 0, ±*π*/2, the fixed point is neutrally stable, when 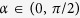, this point is an unstable repellor, and all orbits will evolve to the boundary as a limit cycle that physically corresponds to the in-phase state defined as


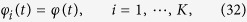


shown in Fig. 2(c).

In order to investigate the in-phase state intuitively, as a matter of fact, in the dynamical model given by Eqs (21) and (22) all the leaves are dynamically equivalent, which means that during the evolution of the system, if at some time *t* = *t*_0_ the phase of any two leaves are equal, then the two leaves will never separate from each other, hence the leaves can never pass each other. The index transformation invariance of Eqs (21) and (22) enables the leaves to be ordered as follows:


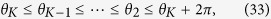


which defines a “canonical invariant region” in phase space^23^, and it should be pointed out that due to the particular symmetry of the system, the canonical invariant region simplifies the structures of the original phase space which makes it possible to seek a low-dimensional description given by equation Eq. (31). On the boundary of the invariant region, the state space has a one-dimensional invariant manifold *M* which corresponds to the in-phase state in the complex ensemble order parameter space, and the invariant manifold *M* is defined by


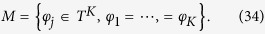


Here *T*^*K*^ is the *K*-dimensional torus^27^. Interestingly, the phase shift *α* plays the role of a dissipation factor for small coupling, and the mean divergence of the phase volume in this invariant manifold is


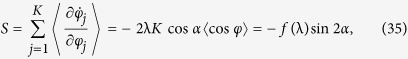


where 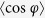 is a time average in one period (we refer the interested reader to the Supplementary Information for the details of the derivations). Hence when 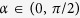, this invariant manifold *M* is attracting. In fact, through numerical calculation, in the original phase space Eq. (23), *M* is a limit cycle (only one Lyapunov exponent is zero, and the other *K* − 1 Lyapunov exponents are negative) and the basin of attraction of the limit cycle *M* is global. The order parameter(with the hub) in *M* can be analytically calculated as


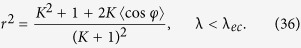


Figure 2(c,d) show the transition from the in-phase state to the synchronous state. As shown in Fig. 2(c) the in-phase state is a limit cycle and the synchronous state is a fixed point on this limit cycle. The transition from the in-phase state to the synchronous state takes place continuously through a saddle-node bifurcation when the coupling strength is increased, as shown in Fig. 2(d).

**Figure 3:**
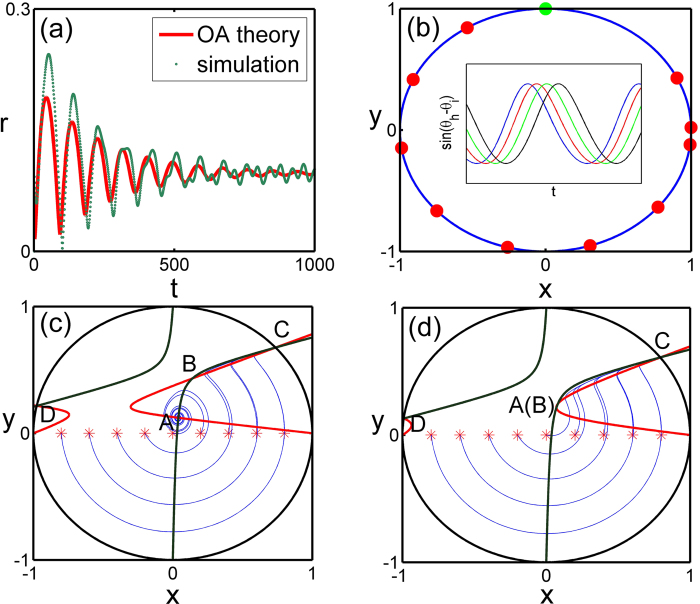
(**a**)Time evolution of the ensemble order parameter with *α* = −0.1*π*, λ = 1.5. (**b**) The stable phase distribution of (**a**) with the reference of the hub, the green is *θ*_*h*_ and the red is *φ*_*j*_, the insert is sin*φ*_*i*_(*t*). The ensemble order parameter phase plane for Δ*ω* = 9, *K* = 10, *α* = −0.1*π*, (**c**) λ = 1.8, (**d**) λ = 2.17. Red lines are 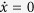, and black 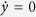. The intersections of them are fixed points A-D. Different initial values with trajectories are marked by ‘*’.

On the other hand, when 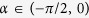, the manifold *M* is unstable and the fixed point in Eq. (31) is a stable attractor, which is physically a splay state. The splay state is the state where phase differences between hub and leaf nodes satisfy


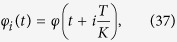


with *T* the period of *φ*(*t*) as shown in Fig. 3(a,b).

In Fig. 3(c,d), we show dynamical manifestations of the discontinuous transition from the splay state to the synchronous state. Fig. 3(c) exhibits the coexistence of the splay state and the synchronous state as the stable fixed points **A** and **C** respectively. The basins of attraction of the splay state and the synchronous state are separated by the repellor **B**. When λ increases, as shown in Fig. 3(d), the repellor **B** and the attractor **A** collide and disappear via an inverse saddle-node bifurcation, and this discontinuous transition makes the synchronous state a global attractor.

The abrupt transition implies that there are two critical coupling strengths 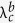 and 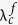, where 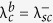 and 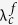 depends on the basins of attraction. The upper limit of 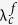 can be determined by analyzing the inverse saddle-node bifurcation as Fig. 1(d)


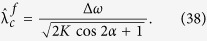


The bistable/coexistence regime corresponding to the above discontinuous transition is given for 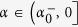 and 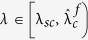.

**Figure 4:**
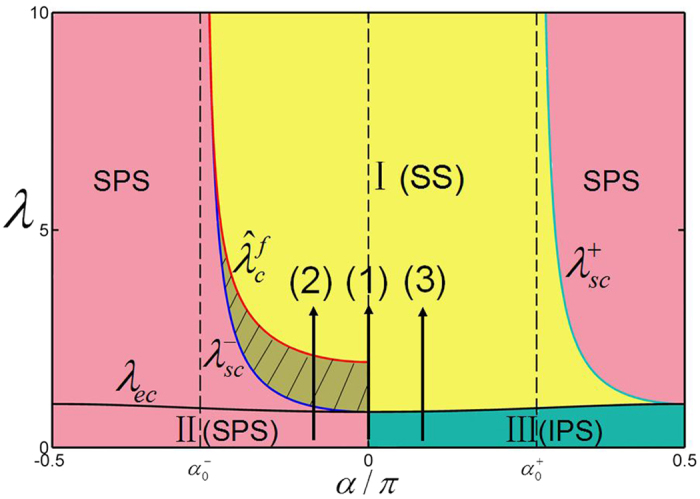
The phase diagram of system Eq. (31). Regime I is the stable synchronous state (SS). Regimes II and III are the asynchronous region with different incoherent states, the splay state (SPS) and the in-phase state (IPS) respectively. The coexistence region of the incoherent state and the splay state is plotted by shadow. Three routes to synchronization are shown as the splay state to the synchronous state, the in-phase state to the synchronous state, and the neutral state to the synchronous state.

A phase diagram describing various dynamical states and transitions of system Eq. (6) is given in Fig. 4, where regime I is the stable region for the synchronous state and regime II for the splay state, regime III for the in-phase state. Three routes from the incoherent state to the synchronous state are shown as (1) the neutral state to the synchronous state, (2) the splay state to the synchronous state, and (3) the in-phase state to the synchronous state. The structure relationship of the incoherent state and the synchronous state determines the feature of the transition.

In addition, the graphical illustration of the linear stability of various steady states with boundaries given by Eqs (27, 28, 29, 30, 31) and the transitions between these states are provided in Fig. 4. The phase shift *α* is divided into four intervals. When 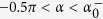, the splay state exists and is always stable for any λ. With the increase of the coupling strength λ unstable synchronous state exists above the threshold λ = λ_*ec*_. When 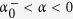, the splay state exists and is stable within 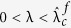, and the synchronous state exists with 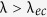 but is unstable unless 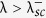. Evidently, there is a co-existing region for the splay state and the synchronous state within the coupling interval 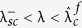. In the third interval where 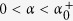, the splay state is always unstable, the stable synchronous state emerges as the coupling strength λ > λ_*ec*_. For the fourth interval 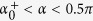, the splay state always exists but is only stable when 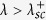, while the synchronous state only exists and is stable in the region 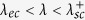. The neutral state exists as a particular case for the phase shift, *α* = 0, ±0.5*π*, and the in-phase state is always stable in the interval 0 < *α* < 0.5*π*, within the coupling range 0 < λ < λ_*ec*_.

### Scale-free network

In the heterogenous network such as the scale-free network, the star graph is a typical topology and is significant for the dynamical process to synchronization^9^,^19^,^28^. Furthermore, in order to reveal the role of the star graphs in the phase transition to synchronization on complex networks, let us consider the dynamical behavior of the networked Kuramomo-like oscillators, the phase of every unit *θ*_*i*_ evolves according to the equation


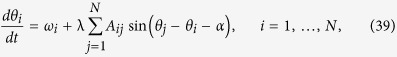


where λ symbols the coupling strength, *ω*_*i*_ the intrinsic natural frequency of the *i*-th oscillator, *α* is the phase shift, *A*_*ij*_ is the elements of of the adjacency matrix *A*, where the elements *A*_*ij*_ = 1 if two nodes *i* and *j* are connected, whereas, *A*_*ij*_ = 0 when nodes *i* and *j* don’t have physical connections. Using Barabasi-Albert model with *m*_0_ = 1^29^ as an example, we generate a scale-free network with *N* = 500 nodes and *K* = 26 nodes in the largest star motif. In addition, considering the character of frequency degree correlation, the intrinsic natural frequency of the node *i* is assigned to be equal to its node degree *k*_*i*_, i.e., *ω*_*I*_ = *k*_*i*_. The above analysis could be applied straightly to studies of the first-order phase transition in the scale-free network qualitatively.

**Figure 5:**
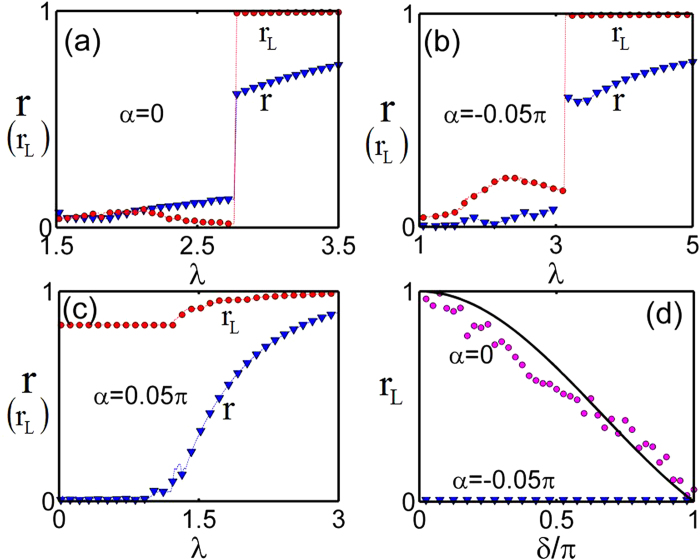
(**a**–**c**): The order parameters of the scale-free network *r* and the largest star motif *r*_*L*_ varies with the coupling strength for different *α*. (**d**) *r*_*L*_ with different initial states randomly chosen from [−*δ*,*δ*] with different *α*, and λ = 0.3, the black solid line is theoretical initial order parameter *r* = sin*δ*/*δ*. The size of network is *N* = 500.

In Fig. 5(a,c), the order parameters of the scale-free network *r* and the largest star motif *r*_*L*_ for three different routes to synchronous state are given. The abrupt transition from the neutral state to the synchronous state and the splay state to the synchronous state are shown in Fig. 5(a,b) respectively, and the continuous transition from the in-phase state to the synchronous state is shown in Fig. 5(c). It is clear that the largest star motif and the scale-free network share the same properties of synchronous behaviors, such as the type of transition, either abrupt or continuous, and the same critical coupling strengthes. Therefore the synchronization transition of the scale-free networks can be well understood in terms of the above discussions on star networks. The original explosive synchronization of the scale-free network^9^ corresponds to the path from the neutral state to the synchronous state, and the property of the neutral state is checked for the largest star motif in the scale-free network in Fig. 5(d), where the order parameters depend on initial conditions.

## Discussion

To summarize, in this paper we proposed the dynamical ensemble order parameter equation in terms of the Ott-Antonsen approach to study the synchronization of coupled oscillators on a star graph. By reducing from a high-dimensional phase space to a much lower-dimensional ensemble order parameter space without additional approximation, one is able to grasp analytically the essential dynamical mechanism of different scenarios of synchronization. Different solutions of the dynamical ensemble order parameter equation such as fixed points and limit cycle build correspondences with different collective states of coupled oscillators, and different bifurcations reveal various transitions among collective states. In the bistable regime, the measure of the synchronous and incoherent states can be analytically obtained by using the dynamical ensemble order parameter equation, which is a very sophisticated and analytically inaccessible procedure in the phase space of coupled oscillators. The analysis and results in the present work can be naturally applicable to scale-free networks, where the star topology plays a dominant role in governing collective dynamics. The properties of three routes to synchronization proposed in star networks are also shown in scale-free networks, which pave the way for analyzing the relation between the star motif and the scale-free network and help us understand the transition to synchronization in more general heterogenous networks.

## Additional Information

**How to cite this article**: Xu, C. *et al.* Explosive or Continuous: Incoherent state determines the route to synchronization. *Sci. Rep.* **5**, 12039; doi: 10.1038/srep12039 (2015).

## Supplementary Material

Supplementary Information
