# Supplementary material for: Explosive or Continuous: Incoherent state determines the route to synchronization
Source: Sci Rep. 2015 Jul 10;5:12039. doi: 10.1038/srep12039 (PMC4498219; doi:10.1038/srep12039)
Supplement: Supplementary Information [file srep12039-s1.pdf]

SUPPLEMENTARY MATERIAL of the Manuscript entitled "Explosive or Continuous: Incoherent state determines the route to synchronization"

Can Xu,[1] Jian Gao,[1] Yuting Sun,[1] Xia Huang,[2] and Zhigang Zheng[1,*]

[1]*Department of Physics and the Beijing-Hong Kong-Singapore Joint Centre for Nonlinear and Complex Systems (Beijing), Beijing Normal University, Beijing 100875, China*

[2]*Department of Mathematics and Physics, North China Electric Power University,Beijing 102206, China*

The mean-field theory of the star graph. In the paper Ref. [18] the authors developed the mean-field theory to solve the Kuramoto model on the star graph with random natural frequency distribution. Based on Eq.(33) and Eq.(34) in their paper, for convenience we rewrite them as below,

$$r^2 = \left(\frac{\Omega - \omega_j}{KJ}\right)^2 + \left[\int_{-J}^{J} d\omega \; g(\omega + \Omega)\sqrt{1 - \left(\frac{\omega}{J}\right)^2}\right]^2, \tag{1}$$

$$\frac{\Omega - \omega_j}{K} = \int_{-\infty}^{\infty} d\omega \; g(\omega + \Omega)\; \omega \left[1 - \sqrt{1 - \left(\frac{J}{\omega}\right)^2}\; \Theta\left(|\omega| - J\right)\right]. \tag{2}$$

As they defined in the paper, where r is the order parameter, Ω is the group velocity, ω_j is the natural frequency of the hub, K is the number of leaves in the star graph, J is the coupling strength. $g(\omega)$ is the distribution function of the natural frequencies of the leaves, Θ is the Heaviside step function. For the particular case of current model, all the leaves are identical which means the distribution function of the natural frequencies takes the form

$$g(\omega) = \delta(\omega - \omega_0), \tag{3}$$

where ω_0 is the natural frequency of the leaf. Substituting Eq.(3) into Eq.(1) and Eq.(2) we obtain a set of two equations for r and Ω

$$r^2 = \left(\frac{\Omega - \omega_j}{KJ}\right)^2 + \left[\int_{-J}^{J} d\omega \; \delta\left(\omega - (\omega_0 - \Omega)\right)\sqrt{1 - \left(\frac{\omega}{J}\right)^2}\right]^2, \tag{4}$$

$$\begin{aligned} \frac{\Omega - \omega_j}{K} &= \int_{-\infty}^{\infty} d\omega \; \delta\left(\omega - (\omega_0 - \Omega)\right)\omega \\ &- \int_{-\infty}^{\infty} d\omega \; \delta\left(\omega - (\omega_0 - \Omega)\right)\omega\sqrt{1 - \left(\frac{J}{\omega}\right)^2}\;\Theta\left(|\omega| - J\right). \end{aligned} \tag{5}$$

Solving Eq.(5) we can obtain the group velocity Ω, then from Eq.(4) we get the order parameter r. The integration above should be discussed in two kinds of cases.
Case I: $|\omega_0 - \Omega| > J$,
one obtains

$$r^2 = \left(\frac{\Omega - \omega_j}{KJ}\right)^2, \tag{6}$$

$$\begin{aligned} \frac{\Omega - \omega_j}{K} &= \omega_0 - \Omega - \omega_0 - \Omega\sqrt{1 - \left(\frac{J}{\omega_0 - \Omega}\right)^2} \\ &= (\omega_0 - \Omega)\left[1 - \sqrt{1 - \left(\frac{J}{\omega_0 - \Omega}\right)^2}\right]. \end{aligned} \tag{7}$$

---

* zgzheng@bnu.edu.cn

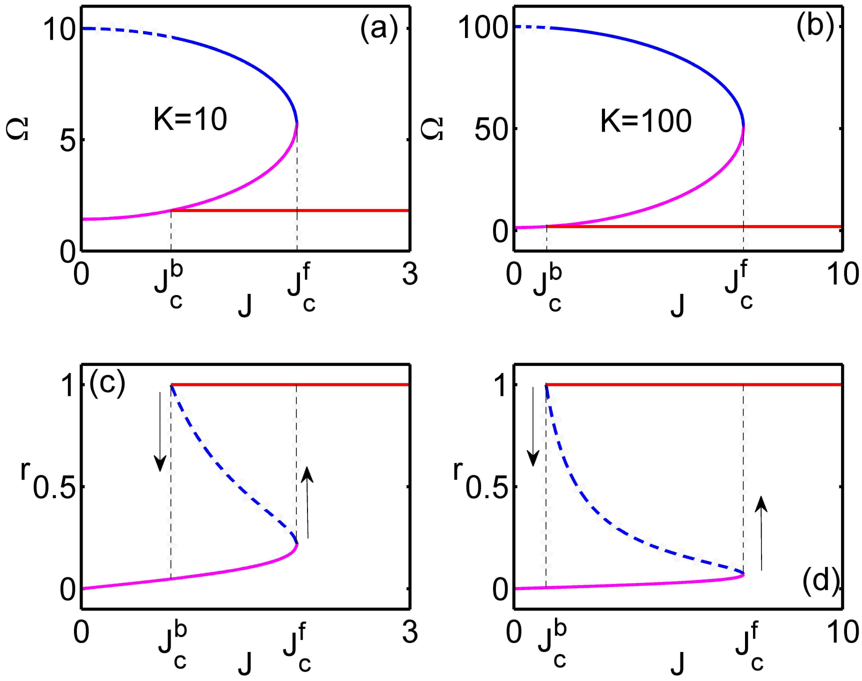

FIG. 1: (a) The group velocity of the star graph with $K = 10$. (b) The group velocity of the star graph with $K = 100$. (c) The order parameter of the star graph with $K = 10$. (d) The order parameter of the star graph with $K = 100$. The dotted line in (a) and (b) is the nonphysical solution which could lead to the order parameter $r > 1$. The dotted line in (c) and (d) is the metastability which could be determined by the linear stability analysis of the dynamical order parameter equation in the paper. For convenience, we set $\omega_j = K$, and $\omega_0 = 1$.

Case II: $|\omega_0 - \Omega| < J$,

$$r^2 = \left(\frac{\Omega - \omega_j}{KJ}\right)^2 + \left[\sqrt{1 - \left(\frac{\omega_0 - \Omega}{J}\right)^2}\right]^2 \\ = 1 + \left(\frac{\Omega - \omega_j}{KJ}\right)^2 - \left(\frac{\omega_0 - \Omega}{J}\right)^2, \tag{8}$$

$$\frac{\Omega - \omega_j}{K} = \omega_0 - \Omega. \tag{9}$$

It is obvious that from Eq.(8) and Eq.(9), we obtain

$$r = 1, \quad \Omega = \frac{K\omega_0 + \omega_j}{K + 1}, \tag{10}$$

which corresponds to the synchronous state. with the decreasing of the coupling strength the synchronous state would lose its stability in the limit case

$$|\omega_0 - \Omega| = J, \tag{11}$$

which means

$$J_c^b = \frac{\omega_j - \omega_0}{K + 1} = \frac{\Delta\omega}{K + 1}. \tag{12}$$

This is the backward critical coupling strength, and the same result could also be found in the main text of the manuscript Eq.(21) with $\alpha = 0$, Ref. [9] and Ref. [21], respectively. Similarly, according to the discriminant of Eq.(7), the group velocity Ω exists within the range $0 \le J \le J_c^f$, where

$$J_c^f = \Big(\frac{\Delta\omega}{\sqrt{K}}\frac{1}{\sqrt{2+K^{-1}}}\Big), \tag{13}$$

is the upper limit of forward critical coupling strength as was shown in Eq.(16) in our paper. Fig. 1 illustrates the group velocity Ω and the order parameter r regardless of the nonphysical solution.

The order parameter in the invariant manifold . In the invariant manifold M, where $\varphi_j = \varphi$, $\forall_j = 1, \cdots, K$, the dynamical equation

$$\dot{\theta}_h = \omega_h + \lambda\sum_{j=1}^{K}\sin(\theta_j - \theta_h - \alpha), \tag{14}$$

$$\dot{\theta}_j = \omega + \lambda\,\sin(\theta_h - \theta_j - \alpha), \tag{15}$$

could be reduced as

$$\dot{\varphi} = \Delta\omega + \lambda\sqrt{(K-1)^2 + 4K\cos^2\alpha}\,\sin(\varphi+\delta), \tag{16}$$

where,

$$\sin\delta = -\frac{(K-1)\lambda\sin\alpha}{b}, \tag{17}$$

$$\cos\delta = -\frac{(K+1)\lambda\cos\alpha}{b}. \tag{18}$$

letting

$$a = \Delta\omega, \tag{19}$$

$$b = \lambda\sqrt{(K-1)^2 + 4K\cos^2\alpha}\,. \tag{20}$$

The average value of $\cos\phi$ in one period reads

$$\langle\cos\varphi\rangle = \int_0^{2\pi}\frac{\cos\varphi\cdot C}{a + b\sin(\varphi+\delta)}d\varphi. \tag{21}$$

The phase difference system Eq.(16) in the invariant manifold M has no fixed point and it is obvious that $a^2 > b^2$, C is a normalizing constant,

$$C = \frac{\sqrt{a^2-b^2}}{2\pi}. \tag{22}$$

Letting $\varphi' = \varphi + \delta$, one obtains the integration of Eq. (21)

$$\langle\cos\varphi\rangle = \int_{-\delta}^{2\pi-\delta}\frac{\cos(\varphi'-\delta)\cdot C}{a + b\sin\varphi'}d\varphi', \tag{23}$$

and it can be calculated by the residue theorem,

$$\begin{aligned}\langle\cos\varphi\rangle &= \frac{(K-1)\lambda\sin\alpha}{b^2}(a - \sqrt{a^2-b^2}) \\ &= \frac{f(\lambda)}{\lambda K}\sin\alpha,\end{aligned} \tag{24}$$

in the interval $0 < \lambda < \lambda_{ec}$, $f(\lambda)$ is a positive value function of λ.
